# Physician and professional caregiver perspectives on meaningful change in agitation behaviors in Alzheimer’s dementia: Insights from qualitative interviews

**DOI:** 10.3389/frdem.2025.1607566

**Published:** 2025-10-10

**Authors:** Jessica Smith, Brian Talon, Ana Martinez, Kelly McCarrier, Jyoti Aggarwal

**Affiliations:** ^1^OPEN Health, Patient-Centered Outcomes, New York, NY, United States; ^2^Lundbeck LLC, Deerfield, IL, United States; ^3^Otsuka Pharmaceutical Development and Commercialization, Inc., Princeton, NJ, United States

**Keywords:** dementia due to Alzheimer’s disease, agitation, neuropsychiatric symptoms, Cohen-Mansfield Agitation Inventory, vignettes, Alzheimer’s disease

## Abstract

**Background:**

Agitation is a common neuropsychiatric symptom of Alzheimer’s dementia. Limited qualitative evidence is available to characterize the clinical meaningfulness of changes in agitation behaviors, as assessed by the Cohen-Mansfield Agitation Inventory (CMAI).

**Objective:**

To collect qualitative data to characterize the magnitude of change in CMAI scores required to represent a clinically meaningful improvement in agitation behaviors from the perspectives of physicians and professional caregivers.

**Materials and methods:**

One-on-one qualitative interviews were conducted with 15 physicians treating Alzheimer’s dementia and 15 professional caregivers. Nine patient vignettes depicting observed changes in CMAI score profiles over a 12-week study period were used as examples of different magnitudes of change in the CMAI total score.

**Results:**

The proportion of participants affirming clinical meaningfulness varied for both physicians and caregivers within and across the nine vignettes presented; however, the four vignettes corresponding to a CMAI total score reduction of 14 or greater were considered clinically meaningful to all participants. Most physicians (8/13) and caregivers (7/13) found a total score reduction of 5 to be clinically meaningful, and some participants (2 caregivers; 0 physicians) articulated that even minimal changes could be clinically meaningful depending on the type of behavior.

**Conclusion:**

Participants who regularly treat people with Alzheimer’s dementia described a significant burden associated with agitation behaviors and provided qualitative examples highlighting that even minor reductions in the frequency of such behaviors can have meaningful benefits for the patient’s care and the burden on professional caregivers and family members.

## 1 Introduction

Agitation is a common neuropsychiatric symptom of dementia due to Alzheimer’s disease (Halpern et al., 2019; Porsteinsson et al., 2014). Symptoms of agitation are diverse (e.g., verbal or physical aggression, socially unacceptable behavior, restlessness, wandering) and are associated with substantial health and financial burdens for patients and caregivers (Halpern et al., 2019; Khoo et al., 2013; Morris et al., 2015; Porsteinsson et al., 2014). Agitation behaviors also represent a social burden through increased financial strain on institutions and increased difficulty in ensuring patient and staff safety in facilities caring for people with dementia due to Alzheimer’s disease (Cloutier et al., 2019; Keszycki et al., 2019). Manifestations of agitation are heterogeneous in presentation, as individuals display unique combinations with various levels of severity (Oberdhan et al., 2024). In people with dementia due to Alzheimer’s disease, agitation negatively impacts their quality of life and cognitive and functional performance and is associated with earlier institutionalization and death (Carrarini et al., 2021; Cloutier et al., 2019; Anatchkova et al., 2019). For caregivers, agitation can cause guilt, distress, and increased caregiving time (Gauthier et al., 2022).

Agitation is a critical target for the development of effective treatments for people with dementia due to Alzheimer’s disease (Antonsdottir et al., 2015). However, few measures are used in clinical practice for the regular assessment of agitation associated with dementia due to Alzheimer’s disease. The Cohen-Mansfield Agitation Inventory (CMAI) is a comprehensive clinician-reported outcome measure assessing the frequency with which 29 key agitation-related behaviors (Table 1) are observed on a scale from 1 (never) to 7 (several times an hour) over a 2-week recall period (Cohen-Mansfield, 1991). The CMAI total score (ranging from 29 to 203) sums the frequency of these behaviors and has been used to support key efficacy endpoints in clinical trials for dementia due to Alzheimer’s disease (Grossberg et al., 2020; Lee et al., 2023). The CMAI is one of the most commonly used tools to assess the frequency of agitation in people with dementia (Cesana et al., 2023). A recent review found statistically significant correlations between the CMAI and other outcomes, including falls/falls risk, patient quality of life, costs/healthcare resource utilization, and caregiver burden (Aggarwal et al., 2023).

**Table 1:** Cohen-Mansfield Agitation Inventory items.

Factor 1: Aggressive behaviors Cursing or verbal aggression Grabbing onto people Tearing things or destroying property Screaming Pushing Hurt self or other (cigarette, hot water, etc.) Throwing things Scratching Kicking Spitting (including at meals) Hitting (including self) Biting Factor 2: Physically non-aggressive behaviors Trying to get to a different place (e.g., out of the room, building) Pace, aimless wandering General restlessness Performing repetitious mannerisms Handling things inappropriately Inappropriate dress or disrobing Factor 3: Verbally agitated behaviors Negativism Complaining Constant unwarranted request for attention or help Repetitive sentences or questions Factor 4: Hiding and hoarding Hiding things Hoarding things Other behaviors Strange noises (weird laughter or crying) Eating/drinking inappropriate substances Intentional falling Making verbal sexual advances Making physical sexual advances

To assist with clinical interpretation of evidence, it is essential to understand what constitutes a meaningful change in agitation. In a qualitative interview study, non-professional caregivers of people with Alzheimer’s disease considered a reduction in the frequency and/or intensity of agitation behaviors captured by the CMAI to reflect meaningful improvement (Oberdhan et al., 2024). Correlations between the CMAI and other patient and caregiver outcomes indicate that a reduction in agitation behaviors may also be associated with meaningful benefits in caregiver burden and in patient and caregiver quality of life (Aggarwal et al., 2023).

A factor analysis using data from the risperidone clinical trial program supported the use of the CMAI total score for the assessment of agitation and aggression in people with Alzheimer’s disease in clinical trials and practice, reporting that it is applicable to a broader range of patients than an assessment based on individual items or domains (Hendrix et al., 2025). While prior research has conducted psychometric validation of the CMAI (Cohen-Mansfield, 1986; Kupeli et al., 2018; Rabinowitz et al., 2005), limited qualitative evidence is available to characterize the clinical meaningfulness of changes in agitation behaviors as assessed by the CMAI. We conducted qualitative interviews with physicians and professional caregivers to characterize their understanding of CMAI score change, including clinical meaningfulness and perceived impact on individuals with dementia due to Alzheimer’s disease and their families and caregivers.

The objective of this study was to explore the magnitude of change in CMAI scores required to represent a clinically meaningful improvement in agitation behaviors from the perspectives of physicians and professional caregivers. In addition, this study aimed to provide context from the clinical perspective of the impact of changes to agitation behaviors on the clinical management of people with dementia due to Alzheimer’s disease. Finally, we aimed to provide context from the observations of physicians and professional caregivers on the impact of changes in agitation behavior on patients’ and caregivers’ lived experiences.

## 2 Materials and methods

### 2.1 Study design

This non-interventional, cross-sectional, qualitative study was conducted using one-on-one semi-structured interviews with physicians and professional caregivers. Prior to data collection, the study protocol was reviewed by Pearl IRB (Indianapolis, IN) and was determined to be exempt from IRB oversight. The study was conducted in accordance with the Declaration of Helsinki. All personal data collected were de-identified and treated as confidential in accordance with applicable local, state, and federal law. Unique participant identification numbers were used to protect participant confidentiality. All study participants provided written informed consent.

### 2.2 Study sample and participant recruitment

The study eligibility criteria were designed to identify a sample of physicians and other professional caregivers who have had opportunities to observe and treat people with dementia due to Alzheimer’s disease and agitation-related features (Table 2). Agitation was defined as behavior involving emotional distress, excessive psychomotor movements, verbal and/or physical aggression, irritability, and poor impulse control, per Cummings et al., 2015.

**Table 2:** Participant inclusion criteria.

Physician cohort	Professional caregiver cohort
US-based physician with current prescribing privileges in active clinical practice Has ≥2 years of clinical practice experience seeing patients with dementia due to Alzheimer’s disease Sees ≥3 patients per month with dementia due to Alzheimer’s disease who display agitation-related behaviors^a^ Willingness to participate in a 45–60-min, audio-recorded, web conference interview	US-based paid caregiver without prescribing privileges (e.g., medical assistant, home health aide, CNA, LPN, or similar) For caregivers working in institutional settings, have seen ≥3 patients with dementia due to Alzheimer’s disease and agitation-related behaviors^a^ per month during the last 6 months For caregivers working in homecare settings, experience with a minimum of 1 such patient will be sufficient, provided that: The caregiver has engaged in a minimum of 10 h of direct patient care per week during the prior 4 weeks, and The caregiver reports working with a minimum of 3 total patients with dementia due to Alzheimer’s disease and agitation during their professional career Willingness to participate in a 45–60-min, audio-recorded, web conference interview

a Agitation is defined as behavior involving emotional distress, excessive psychomotor movements, verbal and/or physical aggression, irritability, and poor impulse control (Cummings et al., 2015).

CNA, certified nursing assistant; LPN, licensed practical nurse; US, United States.

Participant recruitment was managed jointly by the research team and MedPanel, a specialty health sciences recruitment vendor. Potential participants were identified in MedPanel’s healthcare provider databases and contacted via phone or email to provide information about the study. Interested individuals were invited to complete a brief set of screening questions. A sample of 15 physicians and 15 professional caregivers was recruited using purposive sampling (Lasch et al., 2010). A total of 105 clinicians were invited; 31 were screened in and 15 were interviewed. Approximately 350 caregivers were invited; 21 were screened in and 15 were interviewed. Variation in participants’ primary practice settings (community practice vs. institutional/long-term care settings) was pursued, and a target of ≥5 physician participants for each category was achieved. A target of ≥5 professional caregivers who work in community/home care settings was pursued, with the remainder of the cohort working primarily within residential/long-term care settings; this target was not met. In an attempt to recruit a sample that would be representative of healthcare providers treating this population and to capture different perspectives, diversity in clinical specialty (for physicians), age, gender, ethnicity, and geographic location was also pursued throughout recruitment, but no specific target quotas were employed for these characteristics.

### 2.3 Qualitative interviews

Qualitative interviews were conducted by trained interviewers within a web-conferencing platform, in sessions lasting approximately 60 min each. All interviews were audio recorded (with participant consent) and transcribed verbatim for analysis. The interviews were conducted using a semi-structured interview guide (Table 3) in conjunction with nine patient vignettes to explore meaningful changes in agitation behaviors as assessed by the CMAI.

**Table 3:** Overview of interview guide.

Section	Objective	Estimated time	Example questions
1. Introduction	To provide an overview of how the interview will be conducted, reassure confidentiality, and answer any questions	5 min	Do you have any questions before we start? Do you consent to participating in the study and having this interview recorded?
2. History with agitation associated with dementia due to Alzheimer’s disease and the CMAI	To provide the participant with an opportunity to describe their experiences with agitation associated with dementia due to Alzheimer’s disease and describe their familiarity with the CMAI	10 min	How do you define agitation in AD patients? What types of behaviors do you consider agitation? How do you typically evaluate and manage patients with AD who display agitation? Do you use the CMAI or other measures as part of your assessment of agitation in AD patients?
3. Vignettes	To provide the participant with an opportunity to describe their understanding of changes in treating agitation associated with dementia due to Alzheimer’s disease and patient lived experience based on CMAI vignettes	40 min	How would the changes observed in this patient’s CMAI score affect your clinical management of the patient? Do you think the amount of change this patient experienced is clinically meaningful? If yes, why?
4. Closing remarks	To capture any final comments, demographic questions, and thank the participant for their time	5 min	How old are you? What is your gender identity? What race and/or ethnicity do you identify as?
Total time		60 min	

AD, Alzheimer’s disease; CMAI, Cohen-Mansfield Agitation Inventory.

The vignettes used de-identified participant data from recent clinical trials conducted in patients with agitation associated with dementia due to Alzheimer’s disease (NCT01862640, NCT01922258, NCT03548584) and illustrate examples of different magnitudes of change in the CMAI total score (Grossberg et al., 2020; Lee et al., 2023). Each vignette depicted an observed change in an actual clinical trial patient’s CMAI score profile over a 12-week study period (Supplementary Table 1). Vignette data were selected at random from trial data that aligned with nine subgroup categories based on 3 CMAI score change groups (−1 to −5, −6 to −15, −16 to −25) and 3 CMAI baseline score groups (36–60, 61–80, 81–100).

**Figure 1:**
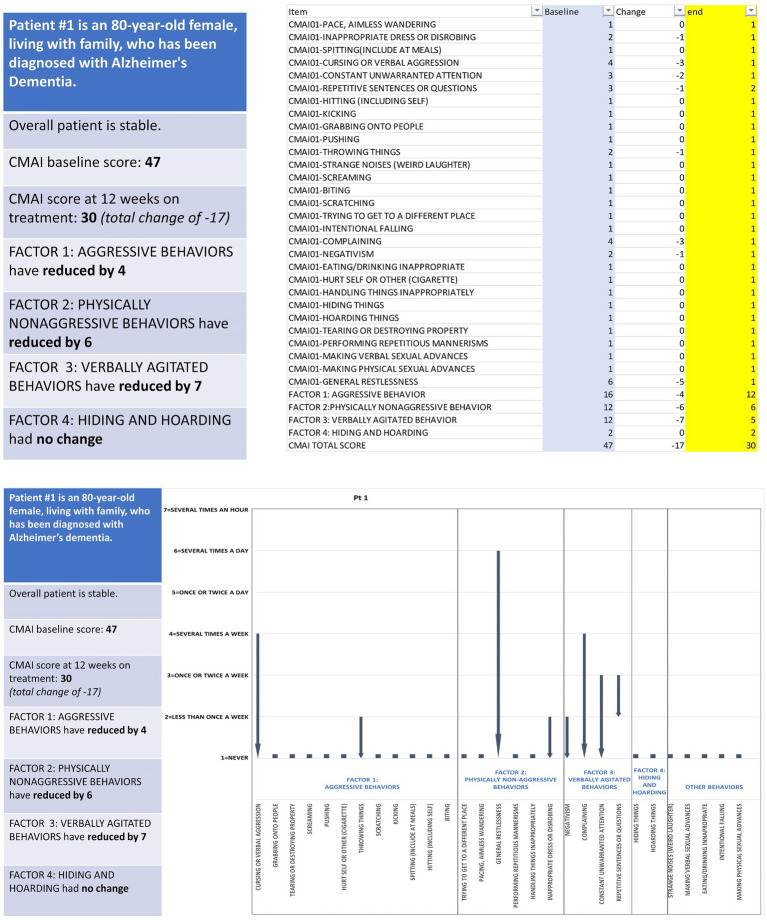
Example patient vignette depicting observed changes in agitation behavior assessed via CMAI. CMAI, Cohen-Mansfield Agitation Inventory.

An example vignette is presented in Figure 1 and describes a stable 80-year-old female with Alzheimer’s dementia who is living with family. After 12 weeks on treatment, the patient had a total CMAI score reduction of 17 points; individual item changes included in this overall score reduction include a decrease in the frequency of cursing or verbal aggression over the CMAI’s 2-week recall period, from several times a week to never (−3 points), and a decrease in general restlessness, from several times a day to never (−5 points). All vignettes were accurate to scores and changes seen in actual trial participants who were otherwise in stable condition, save for their agitation due to Alzheimer’s disease. Patient age, sex, and living situation were selected randomly to be generally representative of the patient population with Alzheimer’s disease. To support clinician and professional caregiver preference in reviewing vignette changes, each vignette was prepared as a numerical (score-based) and visual (graphical) representation of the individual CMAI item and total scores. For the majority of interviews, the vignettes were presented in sequential order (i.e., 1, 2, 3, 4), but due to time constraints, some participants were presented the vignettes in a different order (e.g., 1, 3, 5, 4). The number of vignettes presented to each participant varied based on the pace of their responses and how much time was remaining in the vignette section of the interview.

Interview participants were asked whether they believed the CMAI total score change displayed in each vignette was clinically meaningful, meaningful to the patient, and meaningful to the patient’s family and/or caregivers. However, no vignette was reviewed by all participants due to time constraints. Follow-up probes were asked as needed to gain a deeper understanding of the physician and caregiver perspectives and responses. Upon completion of the approximately 1-h interview, each participant was compensated for their time (physicians: $350; professional caregivers: $150).

### 2.4 Analysis

De-identified interview transcripts were analyzed using ATLAS.ti version 8.0 (ATLAS.ti Scientific Software Development GmbH; Berlin, Germany). Each transcript was coded to identify distinct concepts and themes relevant to characterizing the clinical meaningfulness of changes in CMAI scores. The coding process began with deductive codes drawn from the structure of the CMAI, interview guide, and vignettes. As transcripts were reviewed, new and unanticipated themes emerged and were added to the framework through iterative, inductive analysis.

To ensure coding consistency, three transcripts were dual-coded independently by two researchers and compared to assess inter-coder agreement. Subsequent transcripts were coded independently. After coding was finalized, output from ATLAS.ti was used to characterize the prevalence of selected concepts within the data, specifically types of information sources and elements of participants’ initial descriptions of agitation and responses to vignettes, while the remaining themes were analyzed qualitatively. Supplementary Table 2 provides a condensed overview of our thematic structure, showing each high-level theme and selected associated coded concepts, along with representative quotations.

## 3 Results

### 3.1 Participant characteristics

A total of 30 interviews were completed with physicians (eight neurologists and seven geriatricians) and professional caregivers (13 registered nurses, one licensed practical nurse, and one physician assistant; see Table 4). The study population was predominantly white (60.0%), and the majority of participants self-reported as female (53.3%). Participants ranged in age from 26 to 70 years; the largest age group was 40 to 49 years (33.3%). Multiple study participants indicated that they were currently working in more than one practice setting. The most common practice settings were long-term care (50.0%; mostly independent facilities, 30.0%) and academic/teaching hospitals (43.3%). The majority of study participants (63.3%) had 11 to 25 years of medical practice.

**Table 4:** Self-reported demographic characteristics.

Statistic or category	No. (%) of participants (*n* = 30)
Sex	
Male	9 (30.0)
Female	16 (53.3)
Did not answer	5 (16.7)
Years in practice	
3–5	1 (3.3)
6–10	3 (10.0)
11–25	19 (63.3)
26–30	5 (16.7)
≥31	2 (6.7)
Physician type	
Neurologist	8 (26.7)
Geriatrician	7 (23.3)
Caregiver type	
Physician’s Assistant (PA)	1 (3.3)
Registered Nurse (RN)	13 (43.3)
Licensed Practical Nurse (LPN)	1 (3.3)
Practice setting^a^	
Private practice	7 (23.3)
Hospital system	6 (20.0)
Teaching or academic hospital	13 (43.3)
Long-term care	15 (50.0)
Independent	9 (30.0)
Chain, 2–10 facilities	2 (6.7)
Chain, 11–50 facilities	2 (6.7)
Chain, 51–100 facilities	0 (0)
Chain, ≥100 facilities	1 (3.3)
Race/ethnicity	
White	18 (60.0)
African American / Black	4 (13.3)
Asian (including Indian)	4 (13.3)
Hispanic	2 (6.7)
Did not answer	2 (6.7)
Age group, years	
≤29	1 (3.3)
30–39	6 (20.0)
40–49	10 (33.3)
50–59	5 (16.7)
60+	7 (23.3)
Did not answer	1 (3.3)

a Participants could select more than one response option.

### 3.2 Experience with agitation associated with dementia due to Alzheimer’s disease and CMAI

The most commonly described attributes of agitation in patients with dementia due to Alzheimer’s disease included physically non-aggressive behavior, which broadly aligns with CMAI Factor 2; physically aggressive and verbally aggressive behaviors, which align with CMAI Factor 1; verbally agitated behaviors, which align with CMAI Factor 3; and behaviors unusual for the patient, which do not directly align with the CMAI factor scores (Supplementary Table 3). Three of the 10 participants who noted verbally aggressive behaviors did not mention physically aggressive behaviors. Physicians and caregivers described agitation in other ways not directly aligned with the CMAI, including confusion/delusions/hallucinations (30%), frustration (17%), and distress (10%). Additionally, other specific behaviors such as hyperorality, emotional changes, or increased respiration were indicated by 30% of participants. No participants indicated hiding or hoarding behaviors (CMAI Factor 4) as agitation prior to the presentation of the vignettes.

When participants were asked about their primary source(s) of information about agitation behaviors, physicians and caregivers broadly reported the same types of sources: caregiver (general), family, staff, direct observation of the patient, other sources, and the patients themselves. However, the frequency with which participants reported relying on these sources varied widely between physicians and caregivers. Physicians most frequently relied on information from caregivers (*n* = 11), family (*n* = 7), staff (*n* = 4), and direct observation (*n* = 4). Caregivers most frequently relied on information from family (*n* = 11), staff (*n* = 11), and direct observation (*n* = 8). Caregivers (*n* = 14/15) reported using two or more sources of agitation behavior more frequently than physicians (*n* = 9/15).

The most frequently cited goal for treating agitation behaviors was harm reduction (for the patient, their caregivers [family/staff], and others). Nine physicians and 12 caregivers described harm reduction as a goal of treating agitation associated with dementia due to Alzheimer’s disease. Physicians (*n* = 7) and caregivers (*n* = 4) also described a reduction in the frequency of agitation behaviors as a treatment goal. Increased engagement with family/community or the ability to have patients take part in enrichment activities (such as going shopping, going out to get their hair done, or participating in group activities in long-term care settings) was the third most frequently cited treatment goal (*n* = 3 physicians, *n* = 5 caregivers).

When asked whether they use the CMAI in clinical practice, no physicians or caregivers indicated using the measure. Participants cited the CMAI as being too cumbersome for regular use, even if they thought it provided helpful insights. As part of the screening for this study, participants were asked about their knowledge of the CMAI. Responses ranged from ‘no familiarity and understanding’ (*n* = 1) to ‘great familiarity and understanding’ (*n* = 7) of the CMAI (Supplementary Table 4). The majority of participants indicated having at least a ‘little’ familiarity and understanding of the CMAI (*n* = 15 physicians, *n* = 13 caregivers).

### 3.3 Meaningful change minimums

**Figure 2:**
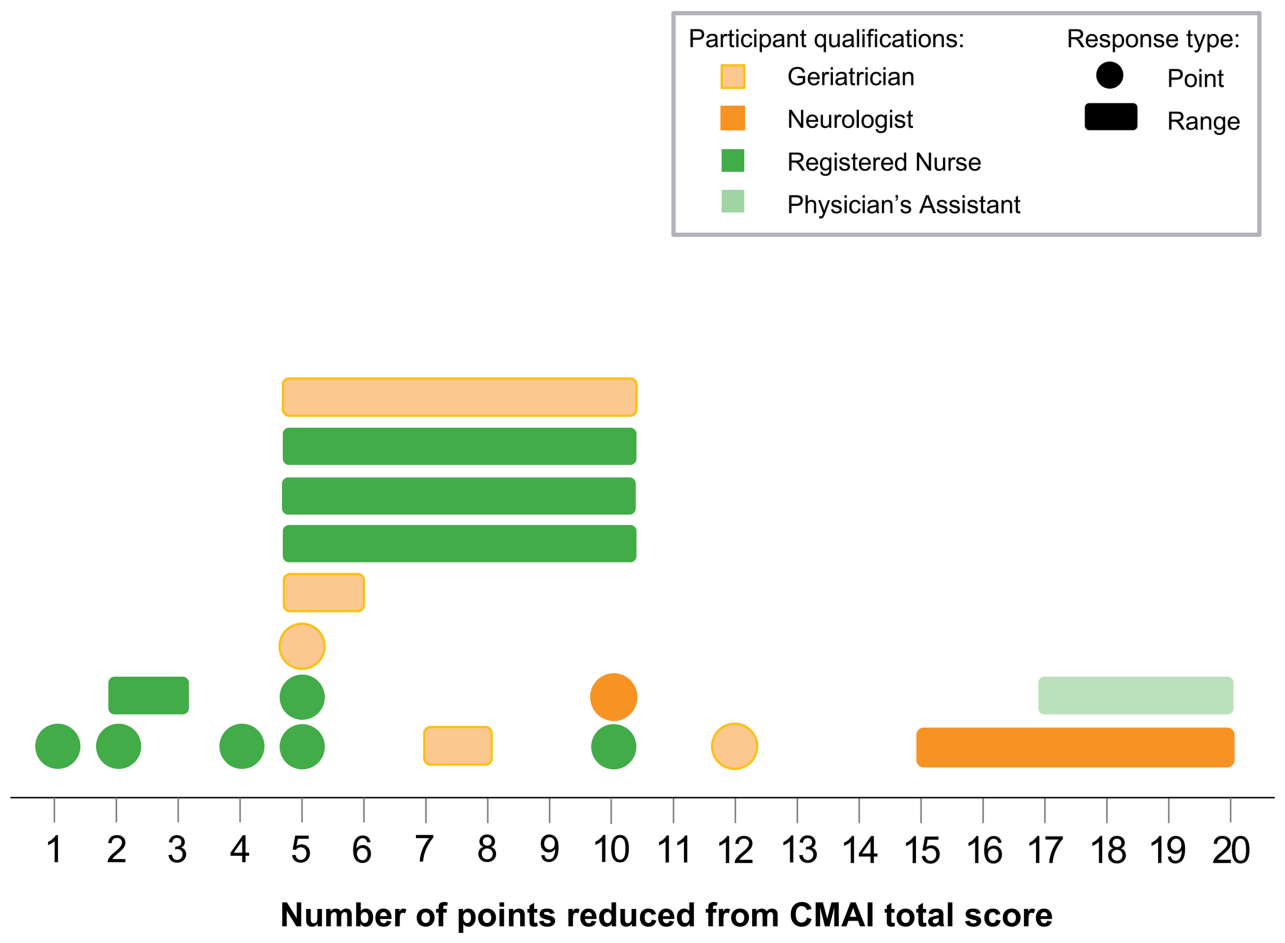
CMAI meaningful change minimums. Each shape represents 1 participant; colors indicate participant qualifications, single-number responses are shown as circles, and point ranges are shown as rectangles. Note: Not all participants provided numerical scores. Several participants provided the percent of change from baseline, while others indicated that the minimum change was dependent on behaviors; these responses are not shown in the figure. CMAI, Cohen-Mansfield Agitation Inventory.

Interviewed physicians and professional caregivers provided a range of responses on the minimum amount of change needed in the CMAI total score for the change to be considered meaningful (Table 5). Of those participants who provided numerical responses, only caregivers (*n* = 4) indicated a minimum change of fewer than 4 points to be clinically meaningful, with one caregiver indicating that changes as low as 1 point in the CMAI total score could be clinically meaningful depending on the types of behavior. Figure 2 shows the distribution of minimum CMAI score changes among interview participants who reported numerical score changes. Both physicians (*n* = 5) and caregivers (*n* = 6) provided responses in the 5- to 10-point range for meaningful change. Three participants (*n* = 2 physicians, *n* = 1 caregiver) indicated a minimum change of more than 11 points for it to be considered clinically meaningful. For those who provided numeric responses, the average minimum change required to be considered meaningful was 9.58 (SD 4.71) for physicians and 6.41 (SD 4.86) for caregivers. Four participants reported the minimum meaningful change as a percentage change from baseline (*n* = 1 caregiver: 5%; *n* = 2 physicians: 10%; *n* = 1 physician: 50%), while six participants indicated that the minimum change was dependent on behavior and did not provide a specific point change.

**Table 5:** CMAI meaningful change minimums by score range.

Range	Representative quotes	Participant #, type, and reported familiarity with CMAI^a^
≤4	**Professional Caregiver 221**: “My thought process there, but that’s looking at, you have to still look at all areas, you know in order to determine that and where in these areas these changes are happening to determine. But I mean, just off the top of my head I say a 4, it may need to be more. But still again even saying the four you have to look at, the behavior changes you know and where they fall in doing that assessment.”	Physicians: NA Caregivers: 202 = RN, 4 206 = RN, 5 218 = RN, 3 221 = RN, 3
5–10	**Physician 110**: “I’m seeing that the score doesn’t necessarily reflect the distress that I’m really looking, you know, really watching out for. And the fact that I need to dive deeper into the scores indicate that I would say you need at least like a 5, more 5 to 10 point change. You know around there to be, you know as convincing as you know, at the minimum I guess and then higher from there would be more convincing. So, I’d say a minimum 5 to 10 base depending on what it’s, what are those leftover symptoms.”	Physicians: 101 = Neurologist, 2 109 = Geriatrician, 3 110 = Neurologist, 1 113 = Geriatrician, 3 119 = Geriatrician, 3 Caregivers: 220 = RN, 1 224 = RN, 3 225 = RN, 2 226 = RN, 5 228 = RN, 2 230 = RN, 4
≥11	**Professional Caregiver 207**: “I guess by at least, you know, 17 to 20 points… I think in order to notice significant improvement in especially like aggressive or verbal symptoms because they’re the most disturbing you, that’s a quiet way to go. And, you know, a dismal, you know, improvement here or there once a week or once a day might be not enough for family to really appreciate or find helpful so that’s why I feel like a, you know a bit more aggressive with treatment as necessary and that’s why I would want to see a more aggressive decrease in numbers, overall numbers from a baseline.”	Physicians: 111 = Geriatrician, 1 116 = Neurologist, 4 Caregivers: 207 = PA, 1
% Change	**Physician 103**: “I would say probably meaningful, minimal is a 10%, you probably need to go by percentage rather than the point because improve 10 point in a patient was a score of 150 point worse is change 10 point in the score of 70 point and the baseline there are different.” **Professional Caregiver 229**: “That’s a, it’s a bit tricky just because you know, it’s almost, it’s really kind of individualized based on which factors are seeing a reduction in the scoring, right? So, you could have somebody who has maybe a 5%, 10% overall score reduction in factors that are less impactful, maybe. It’s hard to quantify, but I would say maybe like a 5% or less than a 5% reduction.”	Physicians: 103 = Neurologist, 2 105 = Neurologist, 1 108 = Geriatrician, 3 Caregivers: 229 = RN, 3
Dependent on behavior	**Physician 115**: “The reason why for me it’s a difficult question is because there’s so many measures that go up and down. You know the impact of the individual measures is important and you know if one stood out and either the family or the staff said this is really what I want to target, I know all these other things are there. And if we, if whatever intervention helps on the specific factors that you’re targeting then before you started the treatment and if they all came down, then that would be significant. And if they said I want to target Factor 1 and 2 and Factors 3 and 4 came down by 15, but Factor 1 and 2 did not change. I don’t think that’s clinically meaningful.” **Professional Caregiver 223**: “I think it all depends on where the patient’s at. I think it depends on what’s been. I don’t think you can go by the totality part of it, I think you have to go by what was decreased and what was increased.”	Physicians: 104 = Neurologist, 3 112 = Geriatrician, 3 114 = Neurologist, 1 115 = Neurologist, 3 117 = Geriatrician, 3 Caregivers: 223 = LPN, 2

^a^ From 1 (most familiarity) to 5 (least familiarity).

CMAI, Cohen-Mansfield Agitation Inventory; LPN, Licensed Practical Nurse; NA, not applicable; PA, Physician’s Assistant; RN, Registered Nurse.

### 3.4 Individual item, one-point change

The majority of participants (*n* = 10/14 physicians and *n* = 10/13 caregivers) indicated that a 1-point change in an individual CMAI item could be clinically meaningful (Table 6). Both physicians and caregivers who considered a 1-point change meaningful often emphasized the importance of reduced behavior frequency in reducing caregiving burden.

**Table 6:** Meaningful change for 1-point change on individual CMAI item.

**n/N (%)**	**Representative quotes**
**Yes**	
Physicians: 10/14 (71.4%)	**104**: “Yeah, it is meaningful but like I said they say but is everything every, every change is important even by a point but that you know, it will not fulfill the satisfaction of the caregivers.” **109**: “That’s an important question. I mean, for me, I’d be okay with the scale change of 1. I take what I can get. And in this case, there are. There are several. Right. There’s several cases. There’s several categories. You know that that went down, and you know one or two points it’s, they should be cumulatively very helpful. Gosh, yeah. It’s a very interesting question. I’m, yeah, I’m putting myself in the shoes of the front line. They’ll take it. They’ll take it for sure. They’ll be happy and thankful and from 7 to 6, but the ticket is an initial success, but I would suspect we’re gonna need more than that degree of change if the baseline is so high.”
Professional caregivers: 10/13 (76.9%)	**221**: “I would. They’re different because something that could happen today could be different tomorrow, you know? But if you see a change that’s pretty constant over a week or so, then that’s significant, going in the right direction, going to what never.” **226**: “Once or twice a week to once a week. I still think the once, the several times an hour down to several times a day, maybe seen as more significant than going from once or twice a week to less than once a week because that was already at a low level. But several times an hour that is so severe, you know, going down to several times a day, that’s so much better. Even though it’s still bad. I still think yes, it would be looked at differently.”
**No**	
Physicians: 4/14 (28.6%)	**103**: “Right, right. I don’t think a one would be any improvement. One is too little.” **116**: “A one point change is very little change in my estimation.”
Professional caregivers: 3/13 (23.1%)	**207**: “Again, I think the meaning meaningful from 7 would be to three.” **228**: “Two weeks. Okay. Well. I’m gonna say. I don’t know that it’s a significant, clinically significant, just one point change, no.”

CMAI, Cohen-Mansfield Agitation Inventory.

### 3.5 Meaningful change: CMAI vignettes

The proportion of participants affirming clinical meaningfulness varied for both physicians and professional caregivers within and across the nine vignettes presented (Table 7). The four vignettes that corresponded to a total score reduction of 14 or more points were considered clinically meaningful to all participants. Most physicians (*n* = 8/13) and professional caregivers (*n* = 7/13) found a total score reduction of 5 points to be clinically meaningful.

**Table 7:** Meaningful change overview by vignette.

Vignette No.	Score change	*n*/*N* (%) of interviewed physicians reporting score change as:			*n*/*N* (%) of interviewed professional caregivers reporting score change as:		
		Clinically meaningful	Meaningful to patient	Meaningful to family/caregiver	Clinically meaningful	Meaningful to patient	Meaningful to family/caregiver
1	−17	13/13 (100.0)	5/8 (62.5)	9/9 (100.0)	14/14 (100.0)	8/10 (80.0)	13/13 (100.0)
2	−5	8/13 (61.5)	3/9 (33.3)	5/8 (62.5)	7/13 (53.8)	3/7 (42.9)	11/14 (78.6)
3	−12	14/14 (100.0)	4/6 (66.7)	7/7 (100.0)	12/13 (92.3)	7/9 (77.8)	11/11 (100.0)
4	−4	5/12 (41.7)	1/7 (14.3)	5/9 (55.6)	3/8 (37.5)	2/5 (40.0)	2/5 (40.0)
5	−1	0/11 (0.0)	0/5 (0.0)	1/6 (16.7)	2/7 (28.6)	0/2 (0.0)	1/4 (25.0)
6	−14	7/7 (100.0)	1/1 (100.0)	3/4 (75.0)	6/6 (100.0)	3/4 (75.0)	2/3 (66.7)
7	−11	4/4 (100.0)	No data	2/2 (100.0)	4/5 (80.0)	0/1 (0.0)	2/3 (66.7)
8	−16	4/4 (100.0)	1/1 (100.0)	2/2 (100.0)	4/4 (100.0)	2/2 (100.0)	3/3 (100.0)
9	−16	4/4 (100.0)	No data	3/3 (100.0)	3/3 (100.0)	No data	1/1 (100.0)

#### 3.5.1 Clinical meaningfulness

For example, a majority of participants (*n* = 8/13 physicians, *n* = 7/13 caregivers) who viewed the score change presented for Vignette 2 (−5 points; Supplementary Table 1) found it to be clinically meaningful. Physician 117 focused on the specific behavior changes displayed in Vignette 2, noting the relationship between importance to the family and clinical meaningfulness:

**117**: “Looking at the negativism, complaining, throwing things, strange noises, disrupting, if the family feels those are important outside, then it's clinically meaningful.” [Geriatrician, 11-25 years’ experience, reported familiarity with CMAI 3 (1 most familiarity to 5 least familiarity)]

In contrast, a minority of participants (*n* = 5/12 physicians, *n* = 3/8 caregivers) who viewed the score change presented in Vignette 4 (−4 points; Supplementary Table 1) found it to be clinically meaningful. Physician 115 focused on the reduction in frequency displayed in Vignette 4, noting:

**115**: “Well, I would think so [that the vignette is clinically meaningful]. We've gone, you know, from those things daily to weekly. So yes, I would think that would be a significant enough drop.” [Neurologist, 16-30 years’ experience, reported familiarity with CMAI 3]

Conversely, caregiver 223 stated that the increase in some behaviors and decrease in others presented in Vignette 4 would make the patient more difficult to care for and, therefore, worse off than prior to starting treatment:

**223**: “No … Not for what the alternative is … I feel like the shifts that have increased are ones that are going to get this patient into a long term care facility sooner than he would have prior to the 12 weeks.” [LPN, 11-25 years’ experience, reported familiarity with CMAI 2]

No physicians (*n* = 0/11) and only two caregivers (*n* = 2/7) who viewed the score change presented for Vignette 5 (−1 point; Supplementary Table 1) found it to be clinically meaningful. Caregiver 226 focused on the reduction in frequency of some Factor 2 behaviors (−2 for trying to get to a different place, −3 for general restlessness, +4 for handling things inappropriately), noting:

**226**: “Because the restlessness went down from several times a day to what is that, once or twice a week. And your wandering, went down to never, even though the overall score was not a big change. I think that's significant clinically meaningful anyway.” [RN, 11-25 years’ experience, reported familiarity with CMAI 5]

In contrast, physician 101 stated that the increase in some behaviors and the decrease in others in Vignette 5 would indicate that the described patient has had essentially no change:

**101**: “Probably not. I mean, it's just because one thing balances out the other … Well, part of the problem always is that we have, you know, one factor coming in and, you know, another factor changing. So, you know, it's a balance of one against the other.” [Neurologist, 26-30 years’ experience, reported familiarity with CMAI 3]

Finally, all participants (*n* = 7/7 physicians, *n* = 6/6 caregivers) who viewed the score change presented for Vignette 6 (−14 points; Supplementary Table 1) found it to be clinically meaningful. A common theme was the importance of reduction in Factor 1 behaviors, as highlighted in responses from physician 104 and caregiver 220:

**104**: “I do believe, especially because, again, the impact on the areas that you described with reduction, especially the aggressive behavior really provides you with insight that there is documentation about the benefit to whatever has been done.” [Neurologist, 26-30 years’ experience, reported familiarity with CMAI 3]

**220**: “Yes … The aggressive behaviors have really, really decreased, reduced by 11. That was the bulk of the change. So the aggressive behavior component of this is really working.” [RN, 6-10 years’ experience, reported familiarity with CMAI 1]

#### 3.5.2 Meaningfulness to patient

When asked whether the change represented in Vignette 2 would be meaningful for the patient, most physicians (*n* = 6/9) and caregivers (*n* = 4/7) indicated that it would not. For example, physician 116 indicated that the changes would likely not be noticed by the patient, even if they were aware:

**116**: “I doubt that it would. That would depend on how much insight the patient has in this. The patient may express it as if they are able to know this, for example is ‘I don't feel any better. I still feel bad.’ Maybe all that you may get from the patient themselves.” [Neurologist, 11-25 years’ experience, reported familiarity with CMAI 4]

Few physicians (*n* = 1/7) and caregivers (*n* = 2/5) indicated that the change represented in Vignette 4 would be meaningful for the patient. However, caregiver 225 indicated that the changes would that likely be meaningful for the patient and that they would likely be aware of the changing behavior they are displaying:

**225**: “I would think so, since some things have changed so that they might notice some changes there. I know with the hiding and the hoarding they're aware of it … because they have a reason that they're doing it.” [RN, 11-25 years’ experience, reported familiarity with CMAI 2]

All participants (*n* = 5/5 physicians, *n* = 2/2 caregivers) who were asked indicated that the change displayed in Vignette 5 would not be meaningful to the patient. For example, physician 116 indicated that the change may cause the patient to feel worse:

**116**: “No, the patient themselves would have no noticeable impact depending on the kind of day they were having. They may say ‘well whatever treatment this doctor has me on isn't working. I don't see any difference.’ If they're having a bad day, they may say, ‘I think that doctor is giving me something to make me feel worse.’” [Neurologist, 11-25 years’ experience, reported familiarity with CMAI 4]

For Vignette 6, all physicians (*n* = 1/1) and the majority of caregivers (*n* = 3/4) indicated that the change displayed in the vignette would be meaningful to the patient.

#### 3.5.3 Meaningfulness to family or caregivers

A majority of participants (*n* = 5/8 physicians, *n* = 11/14 caregivers) indicated that the family or caregivers of the patient represented in Vignette 2 would find the change meaningful. For example, caregiver 227 said:

**227**: “Yeah, definitely [meaningful for the family] … I think always it's going to affect the caregivers, you know the people surrounding the patient.” [RN, 3-5 years’ experience, reported familiarity with CMAI 2]

A majority of physicians (*n* = 5/9) and a minority of caregivers (*n* = 2/5) indicated that the family or caregivers of the patient represented in Vignette 4 would find the change meaningful. Caregiver 226 highlighted the reduction in trying to reach a different place:

**226**: “I think it would positively affect the family because anytime you got a patient at home that's seeking to get to a different place, the family can't rest while they're constantly worried the patient's gonna get out and get lost or something will get hurt. I see that a lot with patients before they actually come in the nursing home with families trying to manage them at home. They basically do it until they're worn out. You know, so I think of decreasing those particular behaviors is good for the family.” [RN, 11-25 years’ experience, reported familiarity with CMAI 5]

Interestingly, physicians 108 and 114 both indicated that the meaningfulness to family would be due to changes in Factor 1 behaviors in Vignette 4. Physician 108 stated:

**108**: “It might be a slight, but most because of those attributes and changes to Factor 1, some of the aggressive behaviors.” [Geriatrician, 11-25 years’ experience, reported familiarity with CMAI 3]

Few physicians (*n* = 1/6) or caregivers (*n* = 1/5) indicated that the family or caregivers of the patient represented in Vignette 5 would find the change meaningful. Caregiver 224, who indicated that this change would not be meaningful to family/caregivers, noted that changes in behaviors such as screaming and an increase in handling things inappropriately would cause more stress on the family:

**224**: “I see the screaming started. They're more stressed … No, and I'm seeing handling things inappropriately. I can think of how that would stress family members out, whether they don't know how to flush a toilet or just do some basic things, that's more stress for them.” [RN, 11-25 years’ experience, reported familiarity with CMAI 3]

All participants (*n* = 3/3 physicians, *n* = 3/3 caregivers) indicated that the family or caregivers of the patient represented in Vignette 6 would find the change meaningful. For example, caregiver 207 stated:

**207**: “And I think caregiver would, I think caregiver would be really pleased with this level of improvement.” [PA in Neurology, 11-25 years’ experience, reported familiarity with CMAI 1]

### 3.6 Importance and weighting of CMAI behaviors

Participants were asked to list the five most important and five least important CMAI behaviors after reviewing the vignettes (Table 8). Physicians and caregivers provided a variety of responses, including listing CMAI factors rather than individual behavior types. The behaviors of most concern, noted by more than one-third (*n* = 10) of the study participants, were hurt self or others, physical aggression (Factor 1), cursing or verbal aggression, and hitting. The behaviors of least concern, noted by more than one-third (*n* = 10) of the study participants, were hiding and hoarding (Factor 4), negativism, repetitious mannerisms, and complaining.

**Table 8:** Most/least important CMAI behaviors.

Behavior	Most Important, *n*	Least Important, *n*
Hurt self or others	12	
Physical aggression (Factor 1)	11	
Cursing or verbal aggression	11	
Hitting	10	
Pushing	9	
Grabbing	8	2
Biting	7	
Kicking	7	1
Wandering	7	4
Throwing	7	
Screaming	6	1
Tearing or destroying property	4	
Physical sexual advances	4	
General restlessness	3	6
Repetitive sentences	3	9
Scratching	3	1
Complaining	2	11
Handling things inappropriately	2	2
Trying to get to a different place	2	1
Verbal sexual advances	2	
Eating and drinking inappropriate substances	1	1
Inappropriate dress or disrobing	1	1
Intentional falling	1	
Hiding and hoarding (Factor 4)		15
Negativism		13
Repetitious mannerisms		10
Constant unwarranted attention		4
Physically non-aggressive (Factor 2)		2
Verbal agitation (Factor 3)		2

CMAI, Cohen-Mansfield Agitation Inventory.

Physicians and caregivers noted weighting various factors in the CMAI over others in their interpretation of clinical meaningfulness and meaningfulness to patients, families, and daily caregivers, although their responses varied by participant and by vignette (Supplementary Table 5). The weighting of Factor 1 (aggressive behaviors) as more meaningful was the most common theme among physicians and caregivers. Factor 2 (physically non-aggressive) behaviors and Factor 3 (verbally agitated) behaviors were also mentioned as being more heavily weighted for different vignette score changes, whereas ‘other behaviors’ (i.e., those not accounted for in the CMAI factor scores) and Factor 4 (hiding and hoarding) behaviors were generally discussed by physicians as being weighted less. However, several caregivers discussed Factor 4 as being weighted more heavily in a long-term care setting.

### 3.7 Managing agitation associated with dementia due to Alzheimer’s disease

Study participants were asked about the clinical management of patients with agitation due to Alzheimer’s disease during the first section of the interview and during the discussion of the patient vignettes. Physicians and caregivers often provided examples of multiple elements or strategies for managing patients’ agitation behaviors. When participants were asked, “How do you typically evaluate and manage patients with dementia due to Alzheimer’s disease who display agitation?” in the first section of the interview, the most common element of clinical management was medication (*n* = 9 physicians, *n* = 6 caregivers; Supplementary Table 6). Later in the interview, participants were asked how their clinical management of the patients described in the vignettes would change based on the change in CMAI score. Changes in medication were mentioned most often by both physicians and caregivers, although caregivers also focused on patient interactions with people and the environment/setting, their perceived ability or inability to redirect the patient, or more one-on-one care (Supplementary Table 7).

### 3.8 Impact on and changes to daily lives of patients with agitation associated with dementia due to Alzheimer’s disease

In addition to asking if the patient and family/caregivers would find the change represented in the vignettes meaningful, participants were asked about how the changes in CMAI score might impact the daily life of the patient with agitation associated with dementia due to Alzheimer’s disease. Physicians’ and caregivers’ responses varied across vignettes and within each participant group. Caregivers tended to give more detailed responses related to the ways in which the patients’ lives would be impacted by score changes, often noting activities the patient may be able to engage in, ways that patient care would need to be modified to accommodate worsening or improvement in various behaviors, and the relationships between patients and others. Physicians also noted improvement in patient quality of life, but often with less detail, and often noted impacts on the dynamic between patient and caregiver/family.

For example, in Vignette 3, physician 117 briefly described the improvement in the patient’s quality of life and the increased likelihood that the patient would stay at home:

**117:** “So I really think in this patient those numbers, actually are more likely to improve the quality of the life of the patient and interaction of the patient with the caregivers and family, so are more like she's more likely to stay home if that relationship is continued to be better rather than, you know, being cursed out continuously no matter what you do and the family burnout is higher and she's more likely to be placed.” [Geriatrician, 11-25 years’ experience, reported familiarity with CMAI 3]

In contrast, caregiver 228 provided a more detailed description based on the same vignette, noting which behavior changes would impact the patient’s daily life and how these behaviors may have different impacts if the patient lived at home or in a care setting:

**228:** “You know, when you, it's your loved one and they're being physically non-aggressive. They're yelling at you or they're complaining. It's really difficult on you as the caregiver. To get, be the brunt of all of that. I think that's a big plus. Overall, her baseline score was 59, so she's come down 12. I think it's significant for her. I think it's gonna increase not only her functioning but also the relationship there between the two of them … I think we would continue to build on it and again increase activities socialization. All of the things that you know a person like this can hopefully be able to participate in their release planning and their activities and therapy sessions with their loved ones, their family. It's just more likely when they're not, the hiding and the hoarding decreased by one. What was that? It was a four, so now it's a three. And you know that unfortunately would depend in a psych hospital. What are they hiding and hoarding? You have to look at whether it's, you know, something that's dangerous, you know, are they hiding food or are they hiding objects that could be used to hurt themselves or someone else because in the hospital, you know, we have to be aware of what’s other people's access to things as well. But you know, there was a decrease of five and the verbally agitated behavior, which is great. The physically non aggressive behavior a decrease of three the repetitious mannerisms. The aggressive behavior there was only decrease in three, so. That in a facility like ours could you know would have to be managed depending on how the aggressive behavior was the constant unwarranted attention could be trying because you know you have multiple other patients that you also have to contend with, but I think you know it's manageable. It would just be an ongoing process of you know, redirection, distraction, keeping the patient occupied and busy and yes, we've all spent 12 hours answering the same question over and over again, but that's what we do.” [RN, 31+ years’ experience, reported familiarity with CMAI 2]

## 4 Discussion

In this qualitative study, a total of 30 interviews were completed with 15 physicians and 15 professional caregivers. The proportion of participants affirming clinical meaningfulness varied for both physicians and caregivers within and across the nine vignettes presented. The four vignettes corresponding to a total CMAI score reduction of 14 points or greater were considered clinically meaningful to all participants, but there was a wide range of responses regarding the minimum meaningful change in the CMAI total score.

The results of this study provide useful context for interpreting changes in agitation behavior as measured with scales such as the CMAI. Interviewed physicians and professional caregivers both indicated that reductions in CMAI total scores would generally have positive impacts on patients’ daily lives, allowing for more meaningful interactions with family members, caregiving staff, and other residents, as well as the ability to engage in activities that are otherwise prohibited due to agitation behaviors.

Participants noted that minor reductions in the frequency of agitation behaviors (e.g., 1 point on the CMAI) can have meaningful benefits for the patient’s care, the burden on professional caregivers and family members, and patients’ day-to-day experiences. However, this contrasted with participants’ responses to Vignette 5, in which none of the participants considered an overall score change of −1 point to be meaningful. This highlights the importance of understanding the context of the 1-point reduction in the CMAI score. Caregivers noted that an overall score reduction was good, but if it included increases in other subscore areas or individual behaviors, that could actually be much worse for the patient or caregivers. Heterogeneity in the presentation of agitation behaviors suggests the need for a patient-centered approach to agitation management (Keszycki et al., 2019). Nevertheless, our findings are complementary to a previous qualitative study among non-professional caregivers, who considered a reduction in the frequency of agitation behaviors to reflect meaningful improvement (Oberdhan et al., 2024).

Physicians and caregivers also expressed concern about perceived worsening. Both groups of participants indicated that this could lead to negative impacts on patients’ daily lives, specifically highlighting caregiver burnout and burden, additional restrictions on enrichment activities, or even an increase in care level or care setting (e.g., moving from home to long-term care, or from long-term care to a locked ward).

When asked how changes in CMAI scores would impact clinical management, physicians provided examples of how shifting behaviors would affect the patient’s caregivers and family, as well as how modifications to treatment regimens (e.g., changing dosage/tolerability, combinations of various treatments) could lead to optimal outcomes, such as increased safety and avoiding changes in care settings. Of note, caregivers’ discussion of clinical management was often more nuanced than that of physicians, as they spend more time in direct contact with similar patients.

At present, there are few ways for clinicians to determine whether changes in an individual patient’s behaviors over time represent a detectable and clinically relevant deterioration or improvement (Cummings, 2025; De Mauleon et al., 2021). Previous studies have reported minimal clinically important difference (MCID) or meaningful within-patient change (MWPC) estimates for the CMAI total score in people with Alzheimer’s disease, which can be used to help clinicians interpret changes, make treatment decisions, and assess treatment response at the individual patient level (Cummings, 2025). In an anchor-based analysis using data from a longitudinal multicenter observational study conducted in France, the estimated MCID for the CMAI total score was −5 points at 1 month and −17 points at 3 months (De Mauleon et al., 2021). In an anchor- and distribution-based analysis using data from randomized controlled trials of risperidone and mirtazapine, which also included an opinion-based approach in which dementia experts were asked to review clinical vignettes describing improvement in agitation symptoms, the MCID for CMAI total score ranged from −4 points over <1 month to −11 points over 1–3 months (Liu et al., 2025). In a *post hoc* analysis of data from the brexpiprazole clinical trial program, Meunier et al. triangulated anchor- and distribution-based methods to report a MWPC threshold for CMAI total score of −20 points, with a threshold range of −15 to −25 points, in patients with agitation associated with dementia due to Alzheimer’s disease (Meunier et al., 2024). The MCID and MWPC estimates reported in these studies are similar to what we observed, in which a CMAI total score change of −14 points or greater is clinically meaningful to all participants; a change of −5 points was clinically meaningful to 62% of physicians and 54% of caregivers.

Our study involved a variety of individuals who provide significant care to people with agitation associated with dementia due to Alzheimer’s disease, including both professional caregivers and physicians. It is the first study of this type and complements the previous literature by expanding the evidence regarding meaningful change in agitation behaviors across different provider types and care settings. However, it is worth noting that no physicians or caregivers reported using the CMAI in clinical practice, saying it was too cumbersome for regular use, even if they thought it provided helpful insights. Nevertheless, professional caregivers expressed interest in using something like the CMAI to track the frequency of agitation behaviors, help keep track of changes, and highlight any potential deterioration to discuss with the patient’s family.

## 5 Conclusion

In this qualitative study, physicians and professional caregivers who regularly treat people with dementia due to Alzheimer’s disease described a significant burden associated with agitation behaviors and provided qualitative examples highlighting that even minor reductions in the frequency of such behaviors (as low as 1 point on the CMAI) can have meaningful benefits for the patients’ care and the burden on professional caregivers and family members. Physicians’ and caregivers’ interpretations of meaningful change were impacted by the heterogeneity of manifested agitation behaviors and the environments in which a person with dementia due to Alzheimer’s disease resides. Participants noted that the meaningfulness of CMAI score reductions was dependent on the patient, the collection of behaviors displayed, and the behaviors in which reductions occurred. Participants indicated that reductions in CMAI total scores would generally have positive impacts on the daily lives of people with dementia due to Alzheimer’s disease, allowing for more meaningful interactions with family, caregiving staff, and other residents, and the ability to engage in activities otherwise prohibited due to agitation behaviors.

## Data Availability

The original contributions presented in the study are included in the article/Supplementary material; further inquiries can be directed to the corresponding author.
