# Supplementary material for: Physician and professional caregiver perspectives on meaningful change in agitation behaviors in Alzheimer’s dementia: Insights from qualitative interviews
Source: Front Dement. 2025 Oct 10;4:1607566. doi: 10.3389/frdem.2025.1607566 (PMC12549559; doi:10.3389/frdem.2025.1607566)
Supplement: Supplementary file 1 [file Table_1.DOCX]

Supplementary Material

**Supplementary Table 1. Vignette Score Change Overviews**

|  | **CMAI at Baseline** | **Score Change** | **CMAI At 12 Weeks** | **Factor 1: Aggressive** | **Factor 2: Physically Nonaggressive** | **Factor 3: Verbally Agitated** | **Factor 4: Hiding and Hoarding** | **Other^a^** |
| --- | --- | --- | --- | --- | --- | --- | --- | --- |
| Vignette 1 | 47 | -17 | 30 | -4 | -6 | -7 | No change | NA |
| Vignette 2 | 83 | -5 | 78 | -5 | No change | -3 | +5 | -2 |
| Vignette 3 | 59 | -12 | 47 | -3 | -3 | -5 | -1 | NA |
| Vignette 4 | 63 | -4 | 59 | -3 | -6 | +3 | +2 | NA |
| Vignette 5 | 57 | -1 | 56 | No change | -1 | -1 | +1 | NA |
| Vignette 6 | 65 | -14 | 51 | -11 | -3 | -1 | +1 | NA |
| Vignette 7 | 87 | -11 | 76 | -7 | -8 | -6 | +6 | +4 |
| Vignette 8 | 81 | -16 | 65 | -8 | -7 | -2 | +1 | NA |
| Vignette 9 | 73 | -16 | 57 | -3 | -2 | -6 | -4 | -1 |

^a^ Includes: Strange noises (weird laughter), Making verbal sexual advances, Eating/drinking inappropriate, Intentional falling, Making physical sexual advances.

CMAI, Cohen-Mansfield Agitation Inventory; NA, not applicable

**Supplementary Table 2. Thematic Structure**

| **Theme** | Selected **a priori** (bolded) and *emergent* (italicized) coded concepts | **Analysis** | **Key Insights** | **Representative Quotes** |
| --- | --- | --- | --- | --- |
| Conceptualizing agitation | **Physically nonaggressive** | Deductive and inductive | Clinicians and caregivers vary in which behaviors they label agitation, often extending beyond the CMAI factors | 110: “Sure, it ranges pretty drastically from you know non-aggressive symptoms to, you know, to more aggressive and more violent, which are obviously more concerning, so have more weight when thinking about. So, we emphasize those upfront asking about any violent behavior towards themselves or anybody else, or is that anyone felt unsafe ever and it can be an awkward conversation. Just sometimes having to talk to them separately. But you know that it's awkward in the patient. There's often you can get, have outburst in the clinic. So, I would say there's a sense of restlessness to it, so a lot of motor symptoms, a lot of repetitive behaviors, asking the same questions over and over is kind of part of Alzheimer's for everybody. I'd say that's more of, you know, all patients, there's a lot that's very common to repeat themselves and then you get like some impulsive behaviors socially inappropriate behaviors kind of childish reverting back to kind of a more immature state and a lot of issues that go along with that hyper orality.”  115: “Agitation in these patients is any behavior that's outside of the norm that a normal person would not do, which may include increased physical activity, verbal activity, anything at all like that that's out of the usual.”  221: “Restless. Cognitive problems, memory. They're acting not like themselves based on how their family members and friends see them before the condition, and then they're pretty agitated. They're restless, they're not calm. It's more on cognitive. It's more really on cognitive problems. Memory: not able to stay or express stuff like that, not able to move in a normal way like a lot of fidgeting, restless behavior, something like that.”  230: “And in the hospital setting these behaviors would be like if they had a dressing, like a surgical dressing on their leg, they would be taking it off, taking the brace off, not wearing it correctly, pulling out IVs, getting out of bed without assistance, not knowing their limitations. And then of course, if they're you know, really confused, then they can also be different, like paranoias where they don't know where they are. They don't trust our hospital staff and that can be challenging and can cause them to get even aggressive and frustrated. Because they're so confused.” |
|  | **Aggressive** |  |  |  |
|  | **Verbally aggressive** |  |  |  |
|  | **Verbally agitated** |  |  |  |
|  | *Agitation: Other* |  |  |  |
|  | *Unusual for patient* |  |  |  |
|  | *Frustration* |  |  |  |
|  | *Delusion / hallucination / confusion* |  |  |  |
|  | *Discomfort* |  |  |  |
| Sources of behavioral information | **Caregiver** | Deductive | Physicians rely more on family and staff reports; caregivers lean on direct observation and team input | 105 “Mostly I rely on patients' caregiver report. I also made some features of agitation during patients' visits. But, again, in most cases, this is our caregivers report”  110 “Usually I'm getting a lot of the information from caregivers, family members, anyone that I can get that from in addition to the patient themselves and pretty much reliant on the family to give me that information.”  116 “Well, it's based on the report I get from other family members and my experience with these patients.”  221 “It's various things. Family is reporting it, staff reporting observation. Other, excuse me. Other patients may say something or you may see a confrontation between patients. Or you know, where you have to intervene and remove one or the other, but you know various things, observation patients, I mean, sorry observation, family members, staff.”  222 “Family members and friends tell me about it, and at the same time I was able to see them.” |
|  | **Family** |  |  |  |
|  | **Observation** |  |  |  |
|  | **Staff** |  |  |  |
|  | **Other** |  |  |  |
|  | **Patient** |  |  |  |
| Importance of behavior type and environment | **Factor 1: Aggressive behaviors** | Deductive and inductive | Aggressive behaviors are weighted most heavily; the impact of non-aggressive and hiding behaviors depends on care setting | 117: “If this patient would be in the nursing home, probably he's gonna end up staying on the medication because of the aggressiveness of the behavior and the risk of pushing somebody who's like, older and frailed and they then the patient itself. Also being at home improving the behavior that's aggressive towards family members would be more likely to allow him to be in his home for longer.”  223: “The patient that's at a long-term care facility that's gonna be a problem with the hoarding and the hiding… We haven't seen the whole picture of what is it doing to the long-term care facility because now that we're hoarding and hiding, are we doing this on just our own stuff or are we going and hoarding and hiding other patient stuff? That’s going to create issues for other patients that's going to come back to her and then start aggressive behavior interactions.”  226: “seeking to get to a different place, the family can't rest while they're constantly worried the patient's gonna get out and get lost or something will get hurt. I see that a lot with patients before they actually come in the nursing home with families trying to manage them at home. They basically do it until they're worn out.” |
|  | **Factor 2: Physically non-aggressive behaviors** |  |  |  |
|  | **Factor 3: Verbally agitated behaviors** |  |  |  |
|  | **Factor 4: Hiding and hoarding** |  |  |  |
|  | **Other behaviors** |  |  |  |
|  | *Location: Home* |  |  |  |
|  | *Location: Long-term care* |  |  |  |
| Thresholds for meaningful change | **Clinical meaningfulness by vignette 1-9** | Deductive | A drop of 14 points or more is universally considered meaningful; smaller changes (5 or even 1 point) can also be viewed as meaningful when key behaviors shift  Perceptions of clinical meaningfulness, meaningfulness to caregivers and family, and to patients may or may not align | *Example quotes for Vignette 1 with a score reduction of -17*  112: “I think it's clinically meaningful… Because it is affecting the quality of the life for the patient and the caregivers.”  101: “It's very hard to say that the patient would notice a change.”  206: “Okay, so a lot of people would say no [this change is not meaningful to the patient], because at some advanced levels, they're so far out of being able to have insight for themselves. That it almost doesn't matter. But I disagree. And I would say, yes, it does deep in their spirit level. And even just in the, like, I talked about this stimulation and the stress level on them. I would say yes, it does.”  117: “Inappropriate dressing is disrupting. I think that's important, but not that important. Throwing things is a major one. Complaining and negativism are the other two that drop significant. I think there's been seen have a good impact on the morale of the caregiver.”  226: “It could have a positive impact on the staff and the family because it's very difficult having to care for patients with behavior issues, so any kind of improvement is always great.” |
|  | **Meaningfulness for caregiver/family vignette 1-9** |  |  |  |
|  | **Meaningfulness for patient by vignette 1-9** |  |  |  |
| One-point item shifts vs total score | **One point change: individual behavior** | Deductive | Most find one-point change on a single high-burden behavior as meaningful, even if the total score change is minimal | 104: “Yeah, it is meaningful but like I said they say but is everything every, every change is important even by a point but that you know, it will not fulfill the satisfaction of the caregivers.”  228: “OK, I would say yes. I mean still, you're seeing some progress. I guess my other thing would be like, okay, seeing that amount of change in a week or a two-week period. So, then that would be another important factor, but I think still if you're moving from 7 to 6, it's still progress, you're still progressing and then you just have to watch. Am I going to go back up or is the patient gonna go back down? So, I would say it's still progress either way. Any way you look at it, however, the patient moves, you're still progressing. Either if it's bad or good, you're progressing.”  103: "Right, right. I don't think a one would be any improvement. One is too little.” |
|  | **One point change: general** |  |  |  |
|  | **One point change: meaningful** |  |  |  |
| Downstream impact and management | **Clinical management by vignette 1-9** | Deductive and inductive | Score changes drive medication adjustments, activity planning, and can delay institutionalization while improving daily functioning and family interactions | 108: “So that's always you know limitation in terms of treatment. So we use a lot more mood stabilizers and with the dose that we used to. That's probably the biggest limitation is the black box warning on some of these medications and some of the cardiovascular, at least theoretically, mortality or consequences... One, if they're hurting other people, but that stops, they're hurting either a staff or or roommates or other clients in the facility, that's you know, sometimes in the ALS in particular, you know, if someone becomes severe enough, they have to, the lesson to leave, you know, which is typically you just can't. So the goal now now have the staffing to really control them. So that's something the goal to say Okay. Can we get the patient controlled enough to stay in the current facility, based on the level of support they have.”  223: “Yes, we usually when it comes to that, we usually will ask like the family, you know, like it's time to go we need to kind of have a like, a calm down kind of moment. We try to redirect the patient into things that we know that they like. We try redirection a lot before we try medication, so yes.”  101: “Yeah, it would change in terms of several things. Number one is, you know, that they need more ongoing care. So, you know, for example, if they have a professional coming in, they're going to need, you know, increase in the number of people that are working. In other words, instead of an eight-hour shift, maybe two-to-eight-hour shifts, it may push you more toward discussing with the family, the need for a locked facility. You know, you're getting more and more toward that point.”  224: “Still would need to be in the long term care facility, but maybe they would be able to participate more in different activities… I think it would have a positive change. You have somebody that maybe had to have somebody watching them a lot more in the home so now we can take her to the activity room and she'll stay there or not try different things for the patient to have a better quality of life… I think we're positive with saying that like you have different things like they can get their hair done in different things in these facilities and now that she's not hitting people, the hairdresser might actually want to take her… Or have to do her toenails or something like that where they couldn't have done that before.”  116: “I think the patient would enjoy a better standard of living in the sense that caregivers at the nursing home would be able to more successfully… change their clothes, help them with going to the bathroom, feeding them. Making sure that they were adequately hydrated and so I think the caregiver's life would be improved pretty significantly with that change." |
|  | **Patient daily life by vignette 1-9** |  |  |  |
|  | **Treatment goals** |  |  |  |
|  | **Treatment goals: caregiver** |  |  |  |
|  | **Treatment goals: frequency** |  |  |  |
|  | **Treatment goals: other** |  |  |  |
|  | *Treatment goals: increase engagement* |  |  |  |
|  | *Treatment goals: independence* |  |  |  |
|  | *Treatment goals: no agitation* |  |  |  |
|  | *Treatment goals: care setting* |  |  |  |
|  | *Treatment goals: harm reduction* |  |  |  |
|  | *Treatment goals: treatment success* |  |  |  |
|  | *Treatment: PRN* |  |  |  |
| Attitudes toward the CMAI | **Familiarity with CMAI** | Deductive and inductive | Participants find the CMAI valuable for its objectivity and potential to support clinical communication and family understanding, while others emphasized challenges with inter-rater reliability and practical use in care settings. Some suggested streamlining for faster use and others adding open-ended sections to better capture behavior context and nuance | 119: “No, actually, you know, I was not very aware of the scale, but I actually, I really like it like after I reviewed it because it's incredibly objective in the way that it's assessing the behaviors and evaluating for improvement.”  202: “No, I think each category probably means something a little bit different depending on you know, where the patient is when they're being evaluated. But I think overall, it gives you a great idea of where patients at. And again, I think, you know, like I said, like the hiding and hoarding to me wasn't as big of a deal as the wandering. But overall, I think it captures everything that could possibly happen and it gives you enough options to read a frequency so knowing how it's set up and designed, I think it's a very helpful tool.”  109: “What goes through my mind, and I wonder if you guys maybe are looking into this is you know if this tool could be administered quickly? You know, picking out the primary factors and let's say if it's available, you know like a mobile device or maybe some sort of AI or some sort of Apple Watch that can very quickly. You know, help determine help provide the doctor that needed data to act. So I guess a refinement of this tool for practical everyday uses in both acute and chronic setting that would resonate with me.”  218: “So I feel that showing them something concrete like this is on paper, so they can say, oh, you're right. You know, I never thought of it that way. Versus me saying, you know, at this time they were doing this and then this other day they were. Doing that so it's just verbal, you know, talk versus that something actually on paper. So I would think that they would believe this more than me just telling them.” |
|  | **CMAI use** |  |  |  |
|  | **CMAI final thoughts** |  |  |  |
|  | *Insights* |  |  |  |

CMAI, Cohen-Mansfield Agitation Inventory.

**Supplementary Table 3. Reported Definition of Agitation by Behavior Type**

| **Behavior type** | **Physician** | **Caregiver** | **Combined** |
| --- | --- | --- | --- |
| Physically nonaggressive | 6 | 7 | 13 |
| Aggressive | 6 | 5 | 11 |
| Verbally Aggressive | 6 | 4 | 10 |
| Verbally agitated | 5 | 5 | 10 |
| Unusual for patient | 5 | 5 | 10 |
| Other | 6 | 3 | 9 |
| Delusion / hallucination / confusion | 4 | 5 | 9 |
| Frustration | 0 | 5 | 5 |
| Discomfort | 1 | 2 | 3 |
| Hoarding and hiding^a^ | 0 | 0 | 0 |

^a^ Hiding and hoarding behaviors were not mentioned spontaneously by any participant, but were included for completeness as they are included in CMAI Factor 4.

CMAI, Cohen-Mansfield Agitation Inventory.

**Supplemental Table 4. Familiarity with CMAI as Reported During Screening**

| **Level of familiarity** | **Physician, n** | **Caregiver, n** |
| --- | --- | --- |
| I have great familiarity and understanding | 2 | 5 |
| I have high familiarity and understanding | 3 | 3 |
| I have some familiarity and understanding | 5 | 6 |
| I have little familiarity and understanding | 3 | 1 |
| I have no familiarity and understanding | 1 |  |
| No response | 1 |  |

CMAI, Cohen-Mansfield Agitation Inventory.

**Supplementary Table 5. Representative Quotes: Weighting Factors for Vignette 2**

| **Participant type** | **Representative quotes** |
| --- | --- |
| Physician | **105**: “So, I would to ask a caregiver. And let's say that I'm a caregiver. Let's imagine that I have somebody to take care of. And for me, of course, **Factor 1**, as depicted here, will be more important than other factors. So **aggressive behavior, probably very important** and other factor is less important from a safety point of view of safety.”  **109**: “I would say **aggressive behaviors**. I mean it's I think it's rightfully Factor 1 because that is you know where you're getting into facility admissions and ER visits and things that are that can be really harmful for the patient to be hospital, admitted to the hospital and, you know, exposed to infections and that sores and what have you so I would say, yeah, that the fact I would rely more on Factor 1 is heavier weight than the others.”  **112**: “Like I said, the physically nonaggressive behaviors or the verbally agitated behaviors don't necessarily bother me this much… But definitely the **physical aggression** definitely impacts it here because and impacts the patient’s safety themselves. So, I pay more attention to those I would say so.”  **114**: “So you know, I'm going to have, you know, kind of a goal of why I'm treating that patient, you know, with medications and of course, the most concerning is going to be the **physically aggressive behaviors,** at least in my mind. And then the verbally agitated ones because that would reflect signs of distress on the patient’s part and we definitely don't want the caregivers or patient to be injured by the physical aggressive behaviors as well. So those would be my 2 priorities.” |
| Caregivers | **207**: “No, you know, with **Factor 1**, I would not weigh that equally with, you know, Factor 4. I think that the risk to suffer, others will always outweigh something that doesn't pose harm to anyone, but simply a nuisance, let's say. So, they cannot be weighted equally.” |

**Supplementary Table 6. Managing Agitation Associated With Dementia Due To Alzheimer’s Disease**

| **Strategy** | **Physician** | **Caregiver** |
| --- | --- | --- |
| Medications | 9 | 6 |
| Environment | 7 | 4 |
| Other health | 4 | 5 |
| Staff engagement | 4 | 6 |
| Redirect^a^ | 4 | 8 |
| Escalation of care (level of care) | 3 | 5 |
| Family / Caregiver (impact on / input from) | 2 | 4 |
| Safety^b^ | 1 | 2 |
| Insurance concerns^c^ | 1 | 0 |
| Observation | 0 | 3 |

^a^ Redirect indicates trying to change the focus of an agitated patient with dementia due to Alzheimer’s disease. This may include giving them a task to do with their hands such as folding towels or engaging them in a conversation on a different topic. The goal is to change the agitation behavior by occupying the patient with dementia due to Alzheimer’s disease through other mental or physical tasks.

^b^ Safety indicates not causing harm to oneself or others.

^c^ One clinician indicated that insurance coverage dictated some of the possible treatments available.

**Supplementary Table 7. Vignette Score Change Prompted Clinical Management**

|  | **Number of Mentions^a^** | |
| --- | --- | --- |
| **Element Of Clinical Management** | **Physicians** | **Caregivers** |
| Medication | 31 | 22 |
| Family / Caregiver (impact on / input from) | 14 | 7 |
| Alternative treatment (non-pharma) | 5 | 4 |
| Level of Care / Supervision | 5 | 14 |
| Relocate | 2 | 3 |
| Difficulty for provider | 2 | 0 |
| Observe / Monitor | 1 | 9 |
| Engagement / Enrichment | 0 | 6 |

^a^ Mentions counted only once per participant per vignette.
